# Human Cognitive Ability Is Modulated by Aromatase Availability in the Brain in a Sex-Specific Manner

**DOI:** 10.3389/fnins.2020.565668

**Published:** 2020-10-19

**Authors:** Nelly Alia-Klein, Rebecca N. Preston-Campbell, Sung Won Kim, Deborah Pareto, Jean Logan, Gene-Jack Wang, Scott J. Moeller, Joanna S. Fowler, Anat Biegon

**Affiliations:** ^1^Friedman Brain Institute, Icahn School of Medicine at Mount Sinai, New York, NY, United States; ^2^Missouri Institute of Mental Health, St. Louis, MO, United States; ^3^National Institute on Alcohol and Alcohol Abuse, Bethesda, MD, United States; ^4^Neuroradiology Unit, Vall d’Hebron University Hospital, Barcelona, Spain; ^5^New York University, Langone Medical Center, New York, NY, United States; ^6^Stony Brook University School of Medicine, Stony Brook, NY, United States; ^7^Brookhaven National Laboratory, Upton, NY, United States

**Keywords:** aromatase, [^11^C]vorozole, estrogen, testosterone, cognition, PET, amygdala, human brain

## Abstract

The enzyme aromatase catalyzes the final step in estrogen biosynthesis, converting testosterone to estradiol, and is expressed in the brain of all mammals. Estrogens are thought to be important for maintenance of cognitive function in women, whereas testosterone is thought to modulate cognitive abilities in men. Here, we compare differences in cognitive performance in relation to brain aromatase availability in healthy men and women. Twenty-seven healthy participants were administered tests of verbal learning and memory and perceptual/abstract reasoning. *In vivo* images of brain aromatase availability were acquired in this sample using positron emission tomography (PET) with the validated aromatase radiotracer [^11^C]vorozole. Regions of interest were placed bilaterally on the amygdala and thalamus where aromatase availability is highest in the human brain. Though cognitive performance and aromatase availability did not differ as a function of sex, higher availability of aromatase in the amygdala was associated with lower cognitive performance in men. No such relationship was found in women; and the corresponding regression slopes were significantly different between the sexes. Thalamic aromatase availability was not significantly correlated with cognitive performance in either sex. These findings suggest that the effects of brain aromatase on cognitive performance are both region- and sex-specific and may explain some of the normal variance seen in verbal and nonverbal cognitive abilities in men and women as well as sex differences in the trajectory of cognitive decline associated with Alzheimer’s disease.

## Introduction

The last and obligatory step in estrogen biosynthesis in all organs and species is catalyzed by the enzyme aromatase (estrogen synthase, Cyp19 gene product), which converts the androgens androstenedione and testosterone, to the estrogens, estrone, and estradiol (Simpson et al., 2002). In reproductively competent women, the ovary is the primary source of circulating estrogens (Simpson, 2003). In both sexes, a major site of extra-gonadal estrogen synthesis is the brain, and it is characterized by widespread but heterogeneous aromatase availability (Biegon et al., 2010a, 2015; Azcoitia et al., 2011; Takahashi et al., 2018). A recent study revealed some region- and sex-specific associations between aromatase availability in the human brain and personality characteristics (Takahashi et al., 2018). To date, however, there are virtually no *in vivo* human studies testing the relationship between brain aromatase *availability* in the human brain and basic cognitive functions. The potential role of aromatase in cognition is supported by animal studies and studies in women with breast cancer reporting that administration of aromatase inhibitors (AIs) is linked to cognitive dysfunction (Rosenfeld et al., 2018). The most common deficits in women were seen in executive function and verbal episodic memory performance, although the effects of aromatase manipulation on neurobehavioral function in both animals and humans appeared to be sexually dimorphic (Shay et al., 2018). Further support of the role of aromatase in human cognition comes from postmortem studies in humans, which demonstrate region-specific changes in aromatase levels in Alzheimer’s disease (Ishunina et al., 2005; Prange-Kiel et al., 2016), suggesting that aromatase may be implicated in normal as well as pathological variations in learning and memory.

The development and application of positron emission tomography (PET) tracers for aromatase have afforded the ability to measure its availability in different brain regions noninvasively in the living human brain (Biegon et al., 2010a, 2015; Takahashi et al., 2018). With the use of this technology, it has been demonstrated that the regional distribution pattern of [^11^C]vorozole is heterogeneous with the highest levels of aromatase availability found in the thalamus and amygdala. In the present study, PET with [^11^C]vorozole was used to measure aromatase availability in the bilateral amygdala and thalamus of healthy men and women. Blood levels of testosterone and estrogen were also obtained. Participants completed tests of verbal learning and memory and perceptual reasoning in order to explore differing domains in cognitive functioning that utilize both verbal and nonverbal abilities, exploring a sex-specific aromatase–cognition association.

## Materials and Methods

### Participants

The study population comprised 27 healthy adult participants (men, *n* = 12; women, *n* = 15), age 21–67 years. All individuals provided written informed consent prior to study participation in accordance with the Institutional Review Board and the Radioactive Drug Research Committee of Stony Brook University/Brookhaven National Laboratory. Participants were excluded for (1) recent or current use of gonadal steroids (including hormonal contraceptives); (2) cigarette smoking (Biegon et al., 2010b, 2012, 2015), recreational drug use, and medications affecting brain function; (3) neurological, psychiatric, or metabolic disorders; and (4) pregnancy in women. During the screening visit, premenopausal women reported the date of their last menstrual period, and PET scans were scheduled to coincide with the early follicular stage.

*Verification of Hormonal Status on Study Day*: On the day of the PET study, blood samples were obtained and sent to an outside laboratory (ARUP) for measurement of hormone levels. In men, free testosterone and estradiol levels were obtained to exclude hypogonadism. Serum estradiol (E2) concentration was determined by tandem mass spectrometry (TMS). In order to calculate free testosterone (fT), total testosterone and sex hormone binding globulin (SHBG) were measured by quantitative electrochemiluminescent immunoassay. Adult male reference intervals for fT (47–244 pg/ml) and E2 (10.0–42.0 pg/ml) were provided by ARUP. In women, progesterone and luteinizing hormone (LH) were additionally measured to verify the stage of the menstrual cycle as well as menopausal status. Five of the 15 women were postmenopausal, defined as age above 50 and more than 12 months since the last menstrual period by self-report and confirmed by high levels of LH, and low estradiol and progesterone in the postmenopausal range. Reference values for the various hormones supplied by ARUP included the following: LH females: follicular: 2.4–12.6 IU/L; mid-cycle: 14.0–95.6 IU/L; luteal: 1.0–11.4 IU/L; postmenopausal: 7.7–58.5. Estradiol: follicular phase, 27–122 pg/ml; mid-cycle phase, 95–433 pg/ml; luteal phase, 49–291 pg/ml; postmenopausal, <41 pg/ml. Progesterone: cycle days reference interval (ng/ml) 1–6, ≤0.17; 7–12, <1.35; 13–15, ≤15.63; 16–28, ≤25.55; postmenopausal: ≤0.10 IU/L).

### Cognitive Tests

Participants completed the California Verbal Learning Test-Second Edition (CVLT-II) (Kramer et al., 2000) by the standard method. The CVLT-II is an individually administered test assessing episodic verbal learning and memory. It measures recall and recognition of two word-lists containing 16 words each recalled over immediate and delayed memory trials. There are five presentation trials followed by an immediate recall of the first list (A), followed by a one-time presentation and immediate recall of the interference list (B). Measures of free and semantically cued recall are obtained after the trial with List B (Short Delay free or cued recall), followed by a 20-min delay (Long Delay Free or Cued Recall) during which the participant cannot engage in verbal tasks. After the delay, a recognition trial is completed during which the participant is asked to identify the items from List A from a larger list that contains distractor words. Following another 10-min delay, a forced-choice trial is administered. We chose select outcome variables aimed at indexing learning through memory [total recall over the five learning trials of list A (Trials 1–5), Short and Delayed Cued Recall, and Short and Delayed Free Recall] as the primary measures of learning on the CVLT-II (Elwood, 1995).

Participants also completed the Matrix Reasoning subtest of the Wechsler Abbreviated Scale of Intelligence (Wechsler, 1999), which is designed to assess nonverbal abstract problem solving, spatial, and inductive reasoning and is considered a general estimate of nonverbal intelligence. In addition, the word reading subtest of the Wide Range Achievement Test-3 (WRAT-3) (Wilkinson, 1993), an estimate of verbal intelligence also considered to be a valid measure of education level, was administered to ascertain letter and word decoding abilities (Wilkinson, 1993; Manly et al., 2002).

### Positron Emission Tomography Scans

The PET images were acquired over a 90-min period using a whole-body positron emission tomograph (Siemen’s HR1, spatial resolution 4.5 mm × 4.5 mm × 4.8 mm, at the center of field of view). Radiotracer synthesis, image acquisition, and PET data analysis were carried out as previously described (Kim et al., 2009; Biegon et al., 2010a). Briefly, subjects were administered [^11^C]vorozole (3–8 mCi; specific activity >0.1 mCi/nmol at the time of injection) intravenously. A metabolite-corrected arterial plasma input function for [^11^C]vorozole was obtained from arterial blood samples withdrawn every 2.5 s for the first 2 min (Ole Dich automatic blood sampler) and then at 3, 4, 5, 6, 8, 10, 15, 20, 30, 45, 60, and up to 90 min (end of study).

### Image Analysis

**FIGURE 1:**
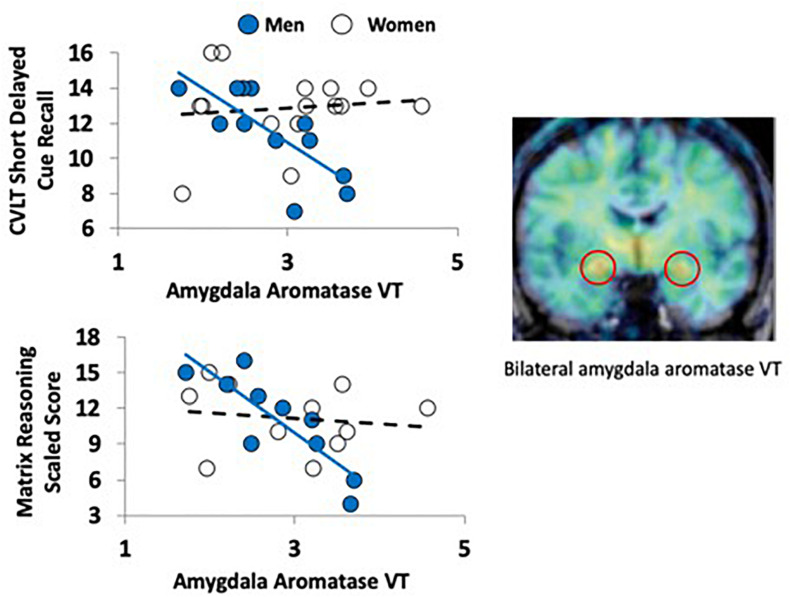
Aromatase availability in the amygdala and cognitive performance in men and women. The PET image on the right (coronal plane, overlaid on MRI) shows the regional distribution of tracer uptake at the level of the amygdala (red circles). The image was pseudocolored using the rainbow scale, with purple/blue at the low end and orange/red at the high end. The graphs on the left show the regression lines between a verbal (top left) and nonverbal (bottom left) task performance and aromatase availability in the amygdala (VT) in men (blue circles) and women (empty circles).

Time frames were summed over the 90-min scanning period. The summed PET images were co-registered with structural three-dimensional magnetic resonance images of the same subject when available, using PMOD software (PMOD Technologies, Zurich, Switzerland) to confirm the anatomical location of tracer accumulation (Figure 1, right panel). Regions of interest (ROIs) were placed bilaterally on the summed image and then projected onto the dynamic images to obtain regional time activity curves. Regions occurring bilaterally (i.e., at a distance from the midline) were averaged. Carbon-11 concentration in each ROI was divided by the injected dose to obtain the % dose/cm^3^. A two-compartment model was used to estimate the total tissue distribution volume, V_*T*_, which includes free and nonspecifically bound tracer as well as specifically bound tracer (Innis et al., 2007). The four model parameters of the two-compartment model were optimized to obtain the best fit to the ROI data for each participant (Flannery et al., 1990; Innis et al., 2007; Biegon et al., 2010a; Pareto et al., 2013; Logan et al., 2014).

### Statistical Analysis

Statistical analyses were conducted using SPSS Statistics software (Version 25, IBM). Independent *t*-tests were used to determine if men and women differed on age, education estimates, cognitive performance, and aromatase availability. The scores on cognitive performance, as well as aromatase availability in the ROIs, were normally distributed; thus, our primary analyses were five general linear models such that sex (categorical), aromatase availability in the amygdala or thalamus (continuous), and their interaction were entered to predict each of five memory outcomes (CVLT-II outcome: Trials 1–5, Long and Short Delay Free and Cued Recall). The same approach was taken for the Matrix Reasoning subtest. A general linear model was used such that sex (categorical), amygdala or thalamus aromatase availability (continuous), and their interaction were entered to predict the estimate of perceptual reasoning. Next, we performed two-tailed Pearson correlations between aromatase availability in the amygdala or thalamus and cognitive performance both across the whole sample and as a function of sex. We further tested the difference in regression slopes between the separate test-by-sex correlations. The cognitive tests use normative data that have been stratified based on age and/or education level(s) as these factors have been found to be consistently correlated with performance, supporting our approach of separate sex by test correlations. Age and education were used as covariates in these analyses, as increasing age and lower education are associated with poorer performance on tasks of verbal learning and memory (Wilkinson, 1993). For education, we used the raw score on the Reading subtest of the WRAT (Manly et al., 2002) as a valid indicator of grade level (Wilkinson, 1993). Finally, we tested whether plasma testosterone and estradiol levels in our sample affected the aforementioned analyses. For this purpose, first, we conducted partial correlations between the cognitive test scores, aromatase availability, and blood measures of estradiol and testosterone. Second, we entered estradiol and testosterone levels in our general linear models to test whether their presence changes the models. All analyses were considered significant at the *p* < 0.05 threshold.

## Results

There were no significant differences between men and women in age, education estimates, and aromatase availability in the amygdala and thalamus (Table 1). Likewise, there were no differences between men and women on their performance on the verbal or nonverbal cognitive tasks (Table 2). However, amygdala aromatase availability correlated with cognitive performance scores (Table 3). Analyses showed that in men only, lower amygdala aromatase availability was associated with better performance on the CVLT-II—recall following a short or long delay (*r* = -0.57 to -0.66, *p* < 0.05). Indeed, the interaction of correlation trend lines, sex × aromatase availability in the amygdala predicted free recall performance following a short delay (free: *F*_1,22_ = 5.76, *p* = 0.025; cued: *F*_1,22_ = 6.51 *p* = 0.018, Figure 1 top left). Similar results were found for recall after a long delay (free: *F*_1,22_ = 4.85, *p* = 0.038) with a trend for Long Delay Cued Recall (*F*_1,22_ = 3.16, *p* = 0.089). The same pattern was revealed on the nonverbal test, Matrix Reasoning (*F*_1,21_ = 5.32, *p* = 0.035) (Figure 1, bottom left). In the thalamus, there were no correlations between aromatase availability and cognitive performance in men or women (men, *r* = -0.08 to 0.30, *p* > 0.35; women, *r* = -0.46–0.25, *p* > 0.09). Plasma levels of estradiol and testosterone did not correlate with aromatase availability in either the amygdala or thalamus, nor with any of the cognitive test scores (*p* > 0.05). Furthermore, adding blood measures of estrogen and/or testosterone to the aforementioned general linear models did not change the results.

**TABLE 1:** Age, reading ability, and aromatase availability in the brains of men and women.

	Test	*p-*value	Male	Female
			(*N* = 12)	(*N* = 15)
Age (years)	t_25_ = -0.56	*p* = 0.58	41.17 ± 16.44	37.53 ± 16.81
WRAT-3 reading subtest	t_20_ = -0.23	*p* = 0.82	101.50 ± 14.01	100.00 ± 16.41
Aromatase VT				
Amygdala	t_25_ = 0.60	*p* = 0.55	2.80 ± 0.60	2.97 ± 0.82
Thalamus	t_25_ = 0.63	*p* = 0.54	4.68 ± 0.86	5.04 ± 1.82

*Values are means ± SD.*

**TABLE 2:** Comparisons between male and female performance on tests of perceptual reasoning and verbal learning and memory.

	Test	*p-*value	Male	Female
			(*N* = 12)	(*N* = 15)
Matrix reasoning^a^	*F*_1_,_8_ = 0.077	*p* = 0.78	10.90 ± 3.90	11.18 ± 2.79
CVLT-II^b^				
Trials 1–5	*F*_2_,_4_ = 2.15	*p* = 0.15	50.75 ± 5.08	54.79 ± 8.09
Short Delay Free Recall	*F*_2_,_4_ = 2.20	*p* = 0.15	10.50 ± 3.00	11.73 ± 2.22
Short Delay Cued Recall	*F*_2_,_4_ = 3.22	*p* = 0.09	11.50 ± 2.43	12.87 ± 2.13
Long Delay Free Recall	*F*_2_,_4_ = 838	*p* = 0.37	11.33 ± 2.64	12.20 ± 2.34
Long Delay Cued Recall	*F*_2_,_4_ = 400	*p* = 0.53	12.17 ± 2.08	12.60 ± 2.35

*Values are means ± SD. ^a^Values for Matrix Reasoning were calculated using Wide Range Achievement Test-3 (WRAT-3) Standard Score as a covariate. ^b^Values for California Verbal Learning Test-Second Edition (CVLT-II) are calculated using age as a covariate.*

**TABLE 3:** Results of Pearson’s correlations between aromatase amygdala availability and measures of abstract reasoning and verbal learning and memory.

	All participants (*N* = 27) *r*	Males only (*N* = 12) *r*	Females only (*N* = 15) *r*
Matrix reasoning^a^	−0.34	−0.84**	−0.08
CVLT-II^b^			
word recall 1–5	−0.17	−0.66*	−0.04
short delay free recall	−0.06	**−0.59^#^**	0.26
short delay cued recall	−0.10	−0.68*	0.15
long delay free recall	−0.11	−0.60*	0.14
long delay cued recall	−0.06	**−0.57^#^**	0.14

*r* = Pearson’s correlation coefficient. ^#^trend (0.1 > *p* > 0.05). *Correlation is significant at the 0.05 level. **Correlation is significant at the 0.01 level. ^a^Values for Matrix Reasoning are calculated using Wide Range Achievement Test-3 (WRAT-3) Standard Score as a covariate. ^b^Values for California Verbal Learning Test-Second Edition (CVLT-II) are calculated using age as a covariate. Bolded values indicate correlations are significant or approaching signifiance.

## Discussion

Here, we have used [^11^C]vorozole, a thoroughly validated radiotracer for brain aromatase (3; 6; 18, 21; 22) in conjunction with PET to examine the relationship between aromatase availability in high-density regions (amygdala and thalamus) and cognitive abilities in healthy subjects. Our data show that brain aromatase availability predicted individual differences in verbal and nonverbal cognitive performance in men but not in women. Men with lower amygdala levels of aromatase had better recall for a list of words (Short Delay Free and Cued Recall and Long Delay Free Recall), on the CVLT-II. Similarly, men with lower aromatase in the amygdala also performed better on the Matrix Reasoning test. These effects were not dependent on plasma levels of estradiol and testosterone.

Animal studies suggest that brain aromatase availability is higher in males than in females and is modulated by changes in testosterone levels (Abdelgadir et al., 1994). As in our previous studies on this cohort (Biegon et al., 2010a, 2015), there were no statistically significant differences in aromatase availability in the amygdala as a function of sex, in line with previous human studies that reported comparable levels of brain aromatase and gene expression in men and women (Steckelbroeck et al., 1999; Stoffel-Wagner et al., 1999). There were also no significant effects of age or hormonal status on aromatase in the brain or any other organ beside the ovary, confirming the organ- and tissue-specific regulation of aromatase expression (Bulun and Simpson, 1994; Biegon et al., 2010a, 2015). Imaging studies suggest that even in the absence of behavioral sex differences, there are clear sex-dependent activations in regions of the brain associated with memory tasks (De Vries, 2004; Gillies and McArthur, 2010), which could be attributed to sex-linked levels of estrogens, androgens, and their receptors in the brain (Cahill, 2006). Therefore, it is not unexpected that despite the absence of sex-based differences in the amygdala aromatase availability and in cognitive performance, the relationship between the two is nonetheless sexually dimorphic.

In this regard, it is important to note that aromatase activity, while giving rise to estrogen, also decreases testosterone levels. In humans, testosterone has been shown to enhance spatial performance in men, whereas estradiol has been shown to enhance verbal memory in women (Matousek and Sherwin, 2010). Furthermore, postmortem studies in brains of men who died with Alzheimer’s disease consistently show large declines in testosterone levels, which correlated with levels of amyloid, a disease pathological marker (Rosario et al., 2004, 2011).

An additional compelling reason to support the sex-specific relationship between aromatase in the amygdala and cognitive function in our cohort, composed of mostly premenopausal women, is that in men, in whom circulating estrogen levels are low, aromatase-dependent production of estrogens from androgens is the main source of estrogens in the brain. This is not true in reproductively competent women, in whom brain levels of estrogen derive from local production as well as peripheral estrogens produced in the ovary, which diffuse freely into the brain. Since ovarian and brain aromatase expression are regulated independently (Rosario et al., 2004) via organ-specific promoter control (Golovine et al., 2003; Rosario et al., 2011), it is to be expected that androgenic and estrogenic modulation of brain function will be regional as well as more tightly correlated with local aromatase availability in men relative to women (Bulun et al., 2003; Golovine et al., 2003).

Our results further suggest that extra-gonadal (i.e., brain) estrogen synthesis and testosterone metabolism, mediated by aromatase, is implicated in verbal and nonverbal cognitive processes and therefore reveals a previously unappreciated sex-dependent relationship between aromatase and cognitive function in humans. Men with lower amygdala levels of aromatase, expected to result in lower estrogen and higher testosterone levels, had better recall for a list of words on the CVLT during short and long delay following encoding. These findings also resonate with a report showing that aromatase inhibition before and during a learning task improved working memory in male rats (Alejandre-Gomez et al., 2007). To date, there have been very few *in vivo* studies of brain aromatase and behavior in humans, yet recently published studies show that individual differences in brain aromatase availability are associated with individual differences in personality traits, with some sex-specific findings (Takahashi et al., 2018; Biegon et al., 2020). This is the first *in vivo* study to show that individual differences in aromatase availability correspond to cognitive performance, including memory. While the amygdala is a brain region best known for modulation of emotion, it is also thought to play a major role in higher cognition (Schaefer and Gray, 2007; Janak and Tye, 2015), and some of these effects are sex-dependent (Cahill, 2010; Carre et al., 2013; Shvil et al., 2014). Based on several studies and theories in the last decades, it is asserted in the literature that amygdala function is implicated in long- and short-term memory, abstract reasoning, and attention vigilance during mentally demanding cognitive tasks such as used in this paper. Even in the absence of emotion triggers during neutral cognitive tasks, attention vigilance and suppression of emotion are needed, implicating the amygdala in the output of every cognitive demand [(for good reviews, see Schaefer and Gray (2007) and Janak and Tye (2015)]. Lastly, robust sex differences have been reported in the functional connectivity of the human amygdala, specifically cortical connections, (Kilpatrick et al., 2006) further suggesting that the effects of varying levels of aromatase in the human amygdala on cognitive function are also likely to be sex-dependent. Notably, we have not observed significant sex difference in verbal learning and memory in our relatively small cohort, although better performance on CVLT in women is a consistent finding across age groups (Kramer et al., 2003; McCarrey et al., 2016; Graves et al., 2017). However, the absolute difference is also consistently small (∼10%, ibid), and studies reporting this difference as significant need a much larger sample (more than 400 subjects/sex, ibid).

## Conclusion

This study used PET with [^11^C]vorozole to document for the first time the association between availability of aromatase in the amygdala and individual differences in cognition in healthy men and women. We demonstrate a clear sex-specific relationship between aromatase levels in the amygdala and cognitive performance, showing that men with lower aromatase availability in the amygdala had better verbal memory and spatial reasoning performance than men having higher amygdala aromatase availability. These findings also suggest that the cognitive impact of brain aromatase is both region- and sex-specific, potentially contributing to the normal variation of cognitive performance in healthy men and women.

## Data Availability Statement

The raw data supporting the conclusions of this article will be made available by the authors, without undue reservation.

## Ethics Statement

The studies involving human participants were reviewed and approved by Institutional Review Board and the Radioactive Drug Research Committee of Stony Brook University/Brookhaven National Laboratory. The patients/participants provided their written informed consent to participate in this study.

## Author Contributions

AB and NA-K conceived the idea and wrote the first draft of the manuscript. All other authors read and edited the final manuscript. In addition, SK was responsible for tracer production. DP and JL performed PET image data analysis and modeling. RP-C conducted statistical analyses and writing. SM assisted in statistical analyses and writing. G-JW was the study physician. JF secured the funding.

## Conflict of Interest

The authors declare that the research was conducted in the absence of any commercial or financial relationships that could be construed as a potential conflict of interest.
